# Negative regulation of receptor tyrosine kinases by ubiquitination: Key roles of the Cbl family of E3 ubiquitin ligases

**DOI:** 10.3389/fendo.2022.971162

**Published:** 2022-07-28

**Authors:** Rong Tang, Wallace Y. Langdon, Jian Zhang

**Affiliations:** ^1^ Department of Nephrology, Xiangya Hospital, Central South University, Changsha, China; ^2^ School of Biomedical Sciences, University of Western Australia, Perth, WA, Australia; ^3^ Department of Pathology, The University of Iowa, Iowa City, IA, United States

**Keywords:** receptor tyrosine kinases, cbl, ubiquitination, negative regulation, degradation

## Abstract

Receptor tyrosine kinases (RTKs) serve as transmembrane receptors that participate in a broad spectrum of cellular processes including cellular growth, motility, differentiation, proliferation, and metabolism. Hence, elucidating the regulatory mechanisms of RTKs involved in an assortment of diseases such as cancers attracts increasing interest from researchers. Members of the Cbl family ubiquitin ligases (c-Cbl, Cbl-b and Cbl-c in mammals) have emerged as negative regulators of activated RTKs. Upon activation of RTKs by growth factors, Cbl binds to RTKs *via* its tyrosine kinase binding (TKB) domain and targets them for ubiquitination, thus facilitating their degradation and negative regulation of RTK signaling. RTKs such as epidermal growth factor receptor (EGFR), platelet-derived growth factor receptor (PDGF), fibroblast growth factor receptor (FGFR) and hepatocyte growth factor receptor (HGFR) undergo ubiquitination upon interaction with Cbl family members. In this review, we summarize the current knowledge related to the negative regulation of RTKs by Cbl family proteins.

## Introduction

Receptor tyrosine kinases (RTKs) are a family of cell surface receptors that participate in morphogenesis, cellular fate processes and pathogenesis. Aberrantly activated RTKs are involved in various diseases such as malignancies and immunological disorders (1, 2). RTKs are tightly regulated by interacting proteins such as ubiquitin ligases. Ubiquitylation of RTKs promotes their trafficking and targeted lysosomal degradation (3, 4). The Casitas B-lineage lymphoma (Cbl) family of proteins (c-Cbl, Cbl-b and Cbl-3) are well-known negative regulators of RTK signaling through their E3 ubiquitin ligase activity (5, 6). Cbl proteins can directly interact with activated RTKs *via* the binding of its Src homology 2 (SH2)-like tyrosine kinase binding (TKB) domain to specific phosphotyrosine peptide motifs of RTKs, leading to the ubiquitination and degradation of RTKs (7). Here, we summarize current knowledge on the negative regulation of RTKs by Cbl family ubiquitin ligases.

## Receptor tyrosine kinases

RTKs are transmembrane proteins that bind extracellular growth factors to control a wide range of cellular processes such as cell growth, motility, proliferation, differentiation and metabolism (1). All RTKs have a similar protein structure consisting of an extracellular ligand binding region, a single transmembrane α-helix, and a cytoplasmic kinase domain that includes a protein tyrosine kinase domain (TKD), a carboxyl (C-) terminal tail plus a juxtamembrane regulatory region (8). Humans possess 58 known RTKs, which are subdivided into 20 different subfamilies based on their variable extracellular ligand binding domain (9, 10). Typical members of this family contain growth factor receptors such as epidermal growth factor receptor (EGFR), platelet-derived growth factor receptor (PDGFR), fibroblast growth factor receptor (FGFR), Nerve growth factor receptor (NGFR), hepatocyte growth factor receptor (HGFR) and colony-stimulating factor 1 receptor (CSF-1R) (11). Besides the insulin receptor (IR) family, other known RTKs are monomers in the membrane of cells (2). Dysregulation or mutation of RTKs and the resultant aberrant activation of downstream signaling pathways have been involved in the development and progression of diseases, such as cancers, immunological disorders and diabetes (12–14).

**Figure 1:**
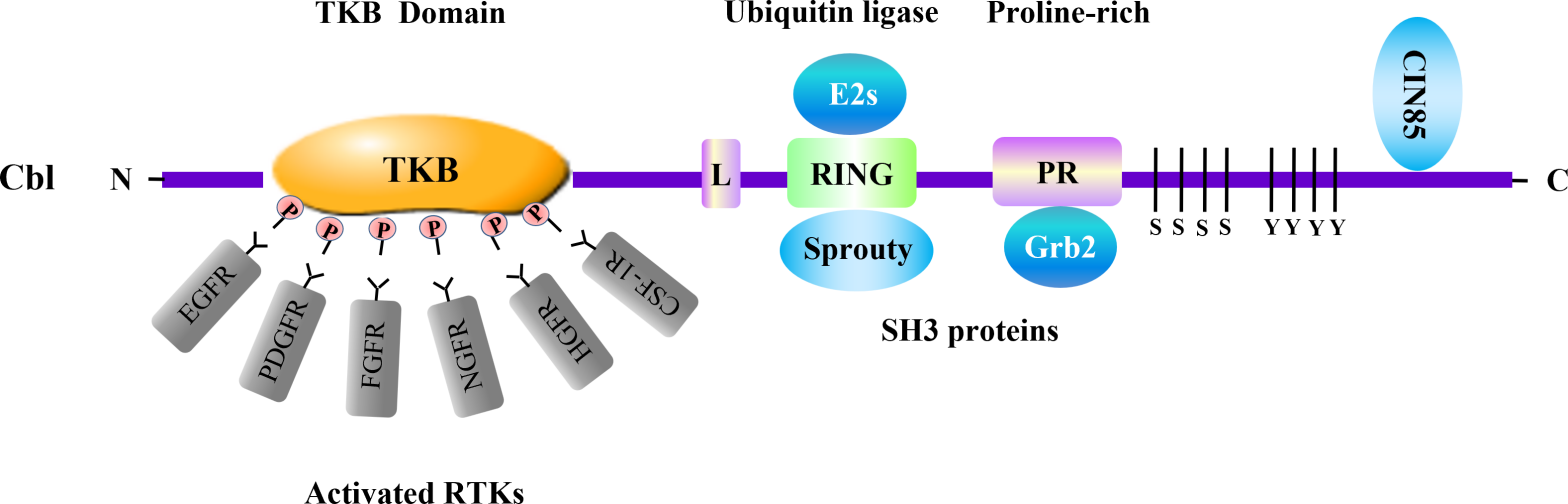
Cbl and adaptor proteins in negative regulation of RTKs. Cbl acts as a ubiquitin ligase involved in negative regulation of multiple RTKs. The Cbl protein consists of several domains which associate with distinct signaling transducers. Among them, the tyrosine kinase binding (TKB) domain binds to various activated RTKs. The RING-finger (RING) domain, which is crucial for the enzymatic activity of Cbl, interacts with adaptor protein Sprouty. Cbl RING finger domain can associate with E2 ubiquitin-conjugating enzymes (E2s) including Ubc4 and UbcH7. The proline-rich region (PR) serves as binding site for SH3-containing proteins such as Grb2. The SH3 domain of CIN85 can interact with the distal carboxyl terminus of Cbl. These molecules are involved in RTKs regulation conducted by Cbl. L, linker.

RTKs are routinely activated by binding receptor-specific ligands to their extracellular regions. Growth factor ligands induce the dimerization and/or oligomerization of RTKs, which leads to autophosphorylation resulting in the recruitment and activation of various downstream signaling proteins and signaling cascades (15, 16). RTKs signaling is negatively regulated by protein-tyrosine phosphatases and ubiquitin ligases. Of the negative regulators, the Cbl family emerges as the most widely studied ubiquitin ligase that associates with RTKs (6, 7) (**Figure 1**).

## Cbl family

As an adaptor molecule and a RING-type E3 ubiquitin ligase, the Cbl family contains three distinct mammalian members, c-Cbl, Cbl-b and Cbl-3 (17). The Cbl family proteins have a conserved N-terminus composed of a tyrosine kinase binding (TKB) domain, an alpha helical linker region and a catalytic RING finger domain. In addition, c-Cbl and Cbl-b contain a less conserved C-terminal region including proline-rich (PR) regions, tyrosine phosphorylation sites and a ubiquitin-associated (UBA)/Leucine zipper (LZ) domain (18). Numerous studies have confirmed that Cbl proteins function as negative regulators in signaling pathways that include RTKs, T cell receptors (TCRs), B cell receptors (BCRs) and C-type lectin receptors (CLRs) that regulate innate and adaptive immune responses (19, 20). Cbl proteins are recruited to activated RTKs through the binding its TKB domain to phosphopeptide motifs produced by receptor autophosphorylation, targeting RTKs for ubiquitylation and degradation by E2 ubiquitin-conjugating (Ubc) enzyme (21–23). The RING domain of Cbl protein recruits the E2 Ubc enzymes including Ubc4, UbcH7, UbcH5B/C, mediating the transfer of ubiquitin from the E2 to the target RTK, synergistically support the ligand-induced ubiquitinationaa (24–26). Hence, Cbl proteins serve as negative regulator of RTKs (**Table 1**).

**Table 1:** Negative Regulation of RTKs by Cbls.

RTK	Ligand	Cbl	Reference
ErbB-1	EGF	c-Cbl, Cbl-b, Cbl-3	(27, 28)
PDGFR-α	PDGF	Cbl	(29, 30)
PDGFR-β	PDGF	c-Cbl, Cbl-b	(31)
FGFR2	FGF	Cbl	(32, 33)
TrkA	NGF	c-Cbl	(34)
Met	HGF	c-Cbl	(35)
CSF-1R	CSF-1	c-Cbl	(36)

## RTK downregulation by Cbl family ubiquitin ligases

### EGFR

The EGFR (also recognized as ErbB1 or HER1) belongs to the ERBB family of RTKs that consists of three additional members including ErbB2/HER-2, ErbB3/HER-3, and ErbB4/HER-4 (37, 38). The EGFR emerges as a typical transmembrane receptor that triggers signaling cascades through ligand-elicited dimerization and tyrosine kinase activation (39). The EGFR possesses diverse ligands such as amphiregulin (AR), betacellulin, epidermal growth factor (EGF), transforming growth factor (TGF) -α and heparin-binding EGF. Aberrant EGFR activation stimulates multiple signaling pathways that mediate cellular dysfunctions and pathologies (40, 41).

ErbB-1 undergoes tyrosine phosphorylation in response to ligand binding, whereas overexpression of c-Cbl can mitigate this effect (27, 28, 42). c-Cbl causes the ubiquitin-dependent degradation and down-regulation of ErbB-1, but not ErbB-3. Through c-Cbl’s direct binding to phosphotyrosine 1045 (pY1045) in ErbB-1’s cytosplasmic domain, c-Cbl and ErbB-1 co-localize in endosomes, targeting ErbB-1 for lysosomal degradation through a ubiquitin-dependent process (43). Similarly, overexpression of Cbl-b or Cbl-3 in Chinese hamster ovary (CHO) cells accelerates removal of overexpressed EGFR from the cell surface, leading to its endocytosis and ubiquitination. Interestingly, alternative splicing of a short peptide of Cbl-3 (Cbl-3S), with a defective SH2 domain, does not influence ubiquitination of the EGFR (43). Thus, all three mammalian members of the Cbl family are implicated in desensitization of the EGFR. Furthermore, an endophilin-CIN85-Cbl complex induces ligand-dependent endocytosis and downregulation of the EGFR (44, 45).

### PDGFR

The PDGF family consists of four polypeptide chains that form five biologically active isoforms: PDGF-AA, PDGF-BB, PDGF-AB, PDGF-CC and PDGF-DD (46). The PDGF ligands exhibit cellular effects by binding to two tyrosine kinase receptors: PDGFR-α and PDGFR-β. PDGFR-α and PDGFR-β bind to different ligands with diverse affinities, and they have similar but different activities (47). Ligand binding promotes dimerization, autophosphorylation and activation of the tyrosine kinase domain in PDGFRs (48, 49).

Stimulation of PDGFRs facilitates phosphorylation of c-Cbl, as well as their physical interaction (29). In turn, c-Cbl overexpression accelerates ligand-induced ubiquitination and subsequent degradation of PDGFR-α and PDGFR-β, as well as inhibiting proliferation and survival dependence by PDGF (50, 51). c-Cbl is able to negatively regulate PDGFR-dependent biological functions, which requires the intact tyrosine kinase binding domain of c-Cbl (30, 31, 50). Both c-Cbl and Cbl-b impact PDGFRβ polyubiquitination and internalization of the PDGFR stimulated by its ligand. Cbl-b together with c-Cbl form a complex which also interacts with PDGFRβ after PDGF-BB stimulation. c-Cbl is unable to bind directly to PDGFRβ, indicating that Cbl-b is essential for the interaction of c-Cbl with PDGFRβ (52).

### FGFR

The mammalian FGF family comprises 22 members which have homologous central protein sequences and structure. FGFs interact with four high affinity cell-surface RTKs designated FGFR1-4 (53–55). Binding of FGFs to FGFRs initiates intrinsic tyrosine kinase activity and multiple downstream signaling cascades, such as RAS-MAP and PI3K-AKT, mediating a wide range of cellular responses (32, 56).

FGFR2 activation recruits c-Cbl binding to the receptor, allowing ubiquitination and proteasome degradation of FGFR2 (33). Thus, c-Cbl mediates down-regulation of FGFR2 and attenuation of FGFR2 signaling, which results in PI3K/Akt attenuation and decreased osteoblast survival triggered by FGFR2 activation. On the contrary, no direct interaction has been observed between c-Cbl and FGFR3 during ubiquitination of FGFR3 (57, 58). Interestingly, a correlation has been found between c-Cbl expression and FGFR3 activity. Overactive FGFR3 mutations yield c-Cbl overexpression, while c-Cbl does not influence FGFR3 expression and ubiquitylation (59).

### NGFR

NGF, a member of the neurotrophin family, is expressed in both the nervous system and peripheral organs (60). NGF/NGFR signaling contributes to the development of the nervous system, angiogenesis and inflammatory diseases. NGF exerts its effects through binding to one of its two receptors: the high-affinity receptor tropomyosin receptor kinase A (TrkA) and the low-affinity receptor p75 neurotrophin receptor (p75) (34, 61). Binding of NGF to its receptors causes receptor dimerization, autophosphorylation and activation, which enhances the phosphorylation of downstream cellular proteins and signal transduction (62).

In response to NGF stimulation, c-Cbl is capable of mediating the internalization, endosomal trafficking, ligand-induced ubiquitination and subsequent lysosomal degradation of TrkA. TrkA ubiquitination and degradation also require direct association between c-Cbl and phosphorylated TrkA (63).

### HGFR

HGF, also known as scatter factor (SF), is mainly expressed in is stromal cells and fibroblasts. Mesenchymal epithelial transition factor (Met) the receptor of HGF, is primarily produced by epithelial cells (64). Following stimulation with HGF, the receptor tyrosine kinase Met is auto-phosphorylated and activated in the cytoplasm, triggering intracellular signaling cascade activation (35, 65).

c-Cbl, but not its oncogenic mutants v-Cbl or 70Z/3 Cbl, targets Met for ubiquitylation and degradation to downregulate HGF/Met signaling (66). c-Cbl is recruited to Met though two mechanisms: direct interaction with the juxtamembrane domain of Met by its TKB domain, and indirect interaction through Grb2 *via* its proline-rich domain (67). After binding to ligand-activated Met, c-Cbl undergoes tyrosine phosphorylation, and ubiquitinates Met through recruiting the endophilin-CIN85 complex, resulting in suppression of signal transduction and biological responses. Inhibition of this complex formation is able to prevent downregulation of Met. Thereby, the endophilin-CIN85-Cbl complex is involved in ligand-dependent Met downregulation (68).

### CSF-1R

CSF-1 serves as a growth factor that participates in the regulation of proliferation, differentiation and survival of mononuclear phagocytes (69). The biological activity of CSF-1 is mediated by its high-affinity cognate receptor CSF-1R encoded by the c-fms proto-oncogene. CSF-1 induces CSF-1R dimerization, resulting in the autophosphorylation of tyrosine residues in the cytoplasmic portion of the CSF-1R, which are the binding sites for Src homology 2 (SH2) containing proteins (36, 70).

c-Cbl down-regulates the CSF-1R by targeting it for polyubiquitination and subsequent enhancement in the endocytic rate, which inhibits macrophage proliferation in response to CSF-1 stimulation (71). Further study has confirmed that activated CSF-1R leads to autophosphorylation of multiple tyrosine residues, such as Tyr973 at its carboxy-terminus. The c-Cbl TKB domain binds to activated CSF-1R at the phosphorylated residue Tyr 973, resulting in CSF-1R signaling cessation (72).

## Adaptor proteins involved in regulation of RTKs by the Cbl family

### CIN85

CIN85, recognized as Cbl-interacting molecule of 85 kDa, is a modular-assembled adaptor protein (73). CIN85 consists of a proline-rich region, three SH3 domains and a coiled-coil region. The SH3 domains of CIN85 bind to the distal carboxyl termini of Cbl and Cbl-b, but not to the proline-rich region. There is no association between CIN85 and Cbl-3 in mammalian cells (74, 75).

Studies have indicated that CIN85 and Cbl family interactions are involved in the regulation of activated RTKs. These associations are further enhanced upon tyrosine phosphorylation of c-Cbl or Cbl-b stimulated after EGF and PDGF activation. CIN85 binding to Cbl-b is essential for internalization of EGFR, however it has no direct influence on receptor ubiquitination stimulated by Cbl-b (44). Further, c-Cbl has been found to mediate RTK endocytosis *via* an interaction with the CIN85-endophilin complex. The Cbl-CIN85-endophilin association induces ligand-stimulation degradation of the EGFR and c-Met. Suppression of the Cbl-CIN85-endophilin complex formation is enough to prevent RTK endocytosis and downregulation, without affecting the ubiquitination function of c-Cbl (45, 68).

### Grb2

Growth factor receptor bound protein 2 (Grb2) is a widely expressed adaptor molecule, which mediates various basic cellular functions and downstream signaling pathways (76). Grb2 consists of a Src homology2 (SH2) domain flanked by N- and C-terminal SH3 domains. The Grb2 SH2 domain associates directly with the tyrosine residues in autophosphorylated EGFR, and Grb2 also binds to other RTKs, such as HGFR (77).

Proline-rich sequences of c-Cbl interact with the SH3 domains of Grb2, which indirectly recruit c-Cbl to EGFRs or Met receptors (78, 79). Grb2 negatively regulates RTKs and downstream signaling, including the Ras pathway, through the recruitment of c-Cbl (80). Tyr1045 mutant EGFR, defective in the c-Cbl docking site, shows reduced ubiquitylation and endocytosis, even under the condition of c-Cbl overexpression. Unexpectedly, an EGFR mutant defective at Tyr1045 (Y1045F) still displays ubiquitination and downregulation, most notably in the presence of c-Cbl overexpression and Grb2, because the Grb2/c-Cbl complex is recruited to Grb2 docking sites on the EGFR (78). Additionally, other studies have shown that Grb2 exerts positive effects on RTK signaling and activates the Ras pathway *via* its interaction with guanine nucleotide exchange factor Sos (16). Hence, Grb2 acts as a double-edged sword in its regulation of RTKs signaling.

### Sprouty2

Sprouty was first discovered in Drosophila as a new antagonist of the FGF signaling pathway. There are four Sprouty isoforms in mammals, which include Sprouty1-4 ( (81). The Sprouty proteins are identified as an additional family of putative signaling regulators. The Sprouty family is shown to inhibit RTKs, specifically by suppressing downstream Ras/Raf/ERK signaling activation induced by growth factors such as FGF, PDGF, NGF and VEGF (82, 83).

Sprouty2 can directly bind to the RING finger domain of c-Cbl, and then remove c-Cbl from activated EGFR. As a result, Sprouty2 abrogates c-Cbl-mediated EGFR internalization and ubiquitylation, thus sustaining downstream receptor signaling (84–87). Other Sprouty homologs, such as Sprouty3 and Sprouty4, do not affect EGFR degradation, although they have the c-Cbl-binding motif (80). Moreover, Sprouty2 interacts with CIN85, and functions at the Cbl/CIN85 interface following EGF stimulation, consequently blocking EGFR downregulation (88).

## Conclusion

Although Cbl family proteins have been investigated widely in immune responses, extensive studies also indicate their crucial role in RTK signaling. Overall, Cbl family proteins interact with activated RTKs *via* binding its TKB domain to phosphopeptide motifs of activated RTKs, leading to RTK ubiquitylation and degradation. Importantly, Cbl family proteins can also recruit adaptor molecules (i.e. CIN85, Grb2 and Sprouty) to RTKs, which participate in the regulation of RTK signaling. The ability of Cbl proteins to interact with diverse RTKs, which are implicated in pathogenesis of varied diseases, indicate this family of proteins as attractive targets for therapeutic intervention. However, further research is still required to fully understand the underlying molecular mechanisms of the Cbl family in the regulation of RTKs, which may provide new clues to clinical applications in the future.

## Author contributions

RT and JZ wrote and edited the manuscript. WL edited the manuscript.

## Acknowledgments

This work is supported by the National Institutes of Health (NIH) grants R01 AI090901, AI121196, and 123253 (to JZ). JZ is a University of Iowa Health Care Distinguished Scholar.

## Conflict of interest

The authors declare that the research was conducted in the absence of any commercial or financial relationships that could be construed as a potential conflict of interest.

## Publisher’s note

All claims expressed in this article are solely those of the authors and do not necessarily represent those of their affiliated organizations, or those of the publisher, the editors and the reviewers. Any product that may be evaluated in this article, or claim that may be made by its manufacturer, is not guaranteed or endorsed by the publisher.
